# Structure–function analysis of the ER-peroxisome contact site protein Pex32

**DOI:** 10.3389/fcell.2022.957871

**Published:** 2022-08-09

**Authors:** Fei Wu, Ida J. van der Klei

**Affiliations:** Molecular Cell Biology, Groningen Biomolecular Sciences and Biotechnology Institute, University of Groningen, Groningen, Netherlands

**Keywords:** peroxisome, endoplasmic reticulum, pex32, PEX11, contact site

## Abstract

In the yeast *Hansenula polymorpha*, the ER protein Pex32 is required for associating peroxisomes to the ER. Here, we report on a structure–function analysis of Pex32. Localization studies of various Pex32 truncations showed that the N-terminal transmembrane domain of Pex32 is responsible for sorting. Moreover, this part of the protein is sufficient for the function of Pex32 in peroxisome biogenesis. The C-terminal DysF domain is required for concentrating Pex32 at ER-peroxisome contact sites and has the ability to bind to peroxisomes. In order to better understand the role of Pex32 in peroxisome biogenesis, we analyzed various peroxisomal proteins in *pex32* cells. This revealed that Pex11 levels are strongly reduced in *pex32* cells. This may explain the strong reduction in peroxisome numbers in *pex32* cells, which also occurs in cells lacking Pex11.

## Introduction

Proteins of the Pex23 family exclusively occur in yeast and filamentous fungi (Jansen et al., 2021). Members of this family contain an N-terminal domain with several predicted transmembrane (TM) helices and a DysF motif at the extreme C-terminus. So far, little is known about the function of both domains. For *Saccharomyces cerevisiae* Pex30 and Pex31, two members of the Pex23 family, a reticulon-like region in the membrane-bound domain was shown to display membrane tubulation activity (Joshi et al., 2016).

All yeast species contain several members of the Pex23 family. *S. cerevisiae* has five (Pex28, Pex29, Pex30, Pex31, and Pex32), while *Hansenula polymorpha* has four (Pex23, Pex24, Pex29, and Pex32) (Jansen et al., 2021). The absence of a member of the Pex23 family generally results in abnormal peroxisome numbers and/or size, explaining why these proteins were designated Pex. Some members also play a role in the formation of lipid bodies (Joshi et al., 2018; Wang et al., 2018) or accumulate at nuclear vacuole junctions (NVJs) (Wu et al., 2020; Ferreira and Carvalho, 2021). The function of Pex23 proteins at NVJs is still unknown.

Although Pex23 family proteins were initially reported to be peroxisomal membrane proteins, recent studies showed that they localize to ER (Mast et al., 2016; Wu et al., 2020). Often, Pex23 proteins accumulate at specialized ER regions, where contact sites are formed with other organelles, such as peroxisomes or the nucleus. These specialized ER regions are also implicated in the formation of pre-peroxisomal vesicles and lipid bodies (Joshi et al., 2018).

We recently showed that *H. polymorpha* Pex23, Pex24, and Pex32, but not Pex29, play important roles in the formation of ER-peroxisome contact sites (Wu et al., 2020). In the absence of HpPex23, HpPex24, or HpPex32, fewer peroxisome-ER contact sites occur, paralleled by a reduction in the average peroxisomal membrane surface and decreased peroxisome numbers (Wu et al., 2020; Yuan et al., 2022). The reduction in peroxisomal membrane surface suggests that contact sites may play a role in the transfer of lipids from ER to peroxisomes to allow organellar expansion. Recent data indicated that the bulk lipid transporter protein Vps13 may contribute to lipid transfer at these contacts (Yuan et al., 2022).

Why peroxisome numbers are decreased in cells lacking Pex23 family proteins is still unknown. Intriguingly, *H. polymorpha pex23* and *pex24* cells have very similar peroxisome abnormalities as *H. polymorpha pex11* cells, namely reduced peroxisome abundance together with an increase in organellar size (Krikken et al., 2009; Yuan et al., 2022). Like in *pex23, pex24,* and *pex32* cells, also in *pex11* cells, peroxisome-ER contact sites are disrupted (Wu et al., 2020), suggesting that Pex23 family proteins at ER together with Pex11 at the peroxisomal membrane are involved in peroxisome-ER contact site formation. Pex11 is an abundant peroxisomal membrane protein (PMP), well known for its role in peroxisome multiplication (Erdmann and Blobel, 1995; Krikken et al., 2009; Carmichael and Schrader, 2022). Intriguingly, *S. cerevisiae* Pex11 was shown to be important for the formation of peroxisome-mitochondria contacts, suggesting that it may be a general contact site resident protein (Mattiazzi Ušaj et al., 2015; Wu et al., 2020).

*H. polymorpha* Pex32 is a crucial Pex23 family protein for peroxisome biogenesis because of absence of Pex32 results in most severe peroxisomal defects (Wu et al., 2020). Here, we investigated the function of different domains of HpPex32. We show that the second transmembrane (TM) helix harbors ER targeting information. The DysF domain has the capacity to associate with peroxisomes but is not essential for the Pex32 function. Unexpectedly, Pex11 levels are very low in *pex32* cells. This may explain why peroxisome numbers are low in *pex32* cells, like in *pex11* cells.

## Results

### The overproduced Pex32 N-terminal domain localizes to the ER, while the C-terminal DysF domain can associate to peroxisomes

**FIGURE 1:**
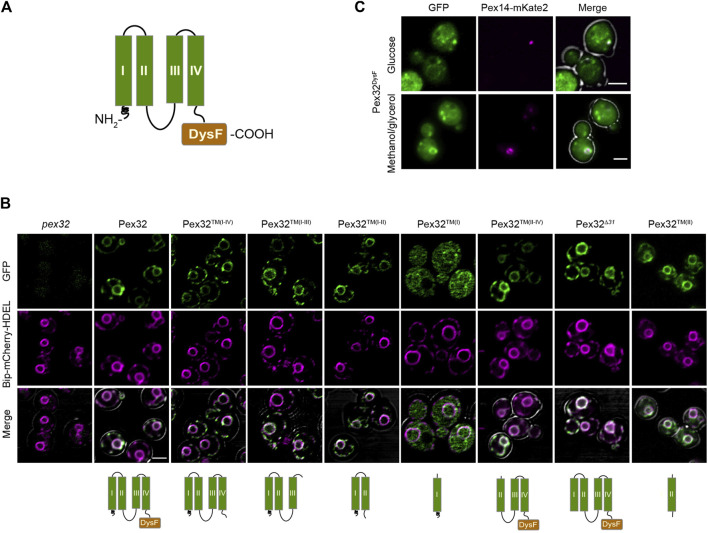
Pex32 TM helices contain ER sorting information, while the DysF domain has the capacity to associate with peroxisomes. **(A)** Predicted Pex32 structure. Transmembrane helixes (TMs) are numbered I, II, III and IV. **(B)** Confocal Laser Scanning Microscopy (CLSM) Airy-scan analysis of glucose-grown pex32 cells producing the ER marker Bip-mCherry-HDEL and the indicated Pex32 truncations fused to GFP and produced under control of the PADH1. Images were processed differently, in order to visualize the GFP signal optimally. Scale bars: 2 μm. **(C)** CLSM Airy-scan images of glucose-grown cells (top) and fluorescence microscopy (FM) images of methanol/glycerol-grown pex32 cells producing Pex14-mKate2 and PADH1Pex32DysF-mGFP (bottom). Scale bars: 2 μm.

Sequence analysis of the *H*. *polymorpha* Pex32 protein revealed four predicted TM helices in the N-terminus and a DysF motif at the extreme C-terminus (Wu et al., 2020) (Figure 1A). To analyze which part of the protein is important for sorting to ER, we constructed several truncated variants containing GFP at the C-terminus. Considering the very low endogenous Pex32 levels (Wu et al., 2020), all truncations were produced under the control of the relatively strong *ADH1* promoter (P_*ADH1*_). We previously showed that overproduced full-length Pex32-GFP localizes to ER similar to the endogenously produced protein (Wu et al., 2020). The constructs were introduced in a *pex32* strain, also producing BiP-mCherry-HDEL as the ER marker, and analyzed by confocal laser scanning microscopy (CLSM, Airy Scan). The western blot analysis using anti-GFP antibodies confirmed that all Pex32 variants were present at the expected molecular weight (Supplementary Figure S1).

In line with our earlier report overproduced full-length Pex32-GFP localizes to the peripheral ER and nuclear envelope [Figure 1B; (Wu et al., 2020)]. Removal of the extreme N-terminal 31 residues, which precede the first predicted TM helix (Pex32^*Δ31*^) or removal of the C-terminal DysF domain (Pex32™^(I−IV)^) did not affect sorting to ER, indicating that ER sorting information is present in the region containing four predicted TM helices, as expected.

To study which region in the membrane-bound domain of Pex32 is required for sorting to ER, the location of several truncated variants was determined by fluorescence microscopy. Of these constructs, a truncation consisting of only the first TM helix [P_*ADH1*_Pex32™^(I)^] was mainly cytosolic, with only very little fluorescence detectable at the nuclear envelope. All other constructs are fully co-localized with the ER marker. These include a construct consisting of only the second TM helix [Pex32™^(II)^], the first second TM helices [Pex32™^(I−II)^] or the first third TM helices [Pex32™^(I−III)^]. Similarly, a construct lacking the first TM helix [Pex32™^(II−IV)^] is localized to the ER. The protein level of a construct consisting of the third and fourth TM helix [without the DysF domain; Pex32™^(III−IV)^] was below the limit of detection by fluorescence microscopy and therefore could not be localized.

To summarize, our study revealed that all constructs containing TM(II) localized to ER, indicating that TM(II) contains ER sorting information. Whether ER sorting information is also present in TM(III) or TM(IV) could not be established.

Overexpression of the soluble DysF domain containing a C-terminal GFP in *pex32* cells (Pex32^DysF^-GFP) resulted in some spots of higher intensity in addition to cytosolic fluorescence (Figure 1C). Co-localization experiments showed that these spots frequently overlapped with the Pex14-mKate2 peroxisomal membrane marker (Figure 1C), indicating that the soluble DysF domain is capable to associate with peroxisomes. Note that not all *pex32* cells contain peroxisomes, therefore many cells lack a Pex14-mKate2 spot (Wu et al., 2020).

### The DysF domain is not essential for Pex32 function in peroxisome biogenesis

**FIGURE 2:**
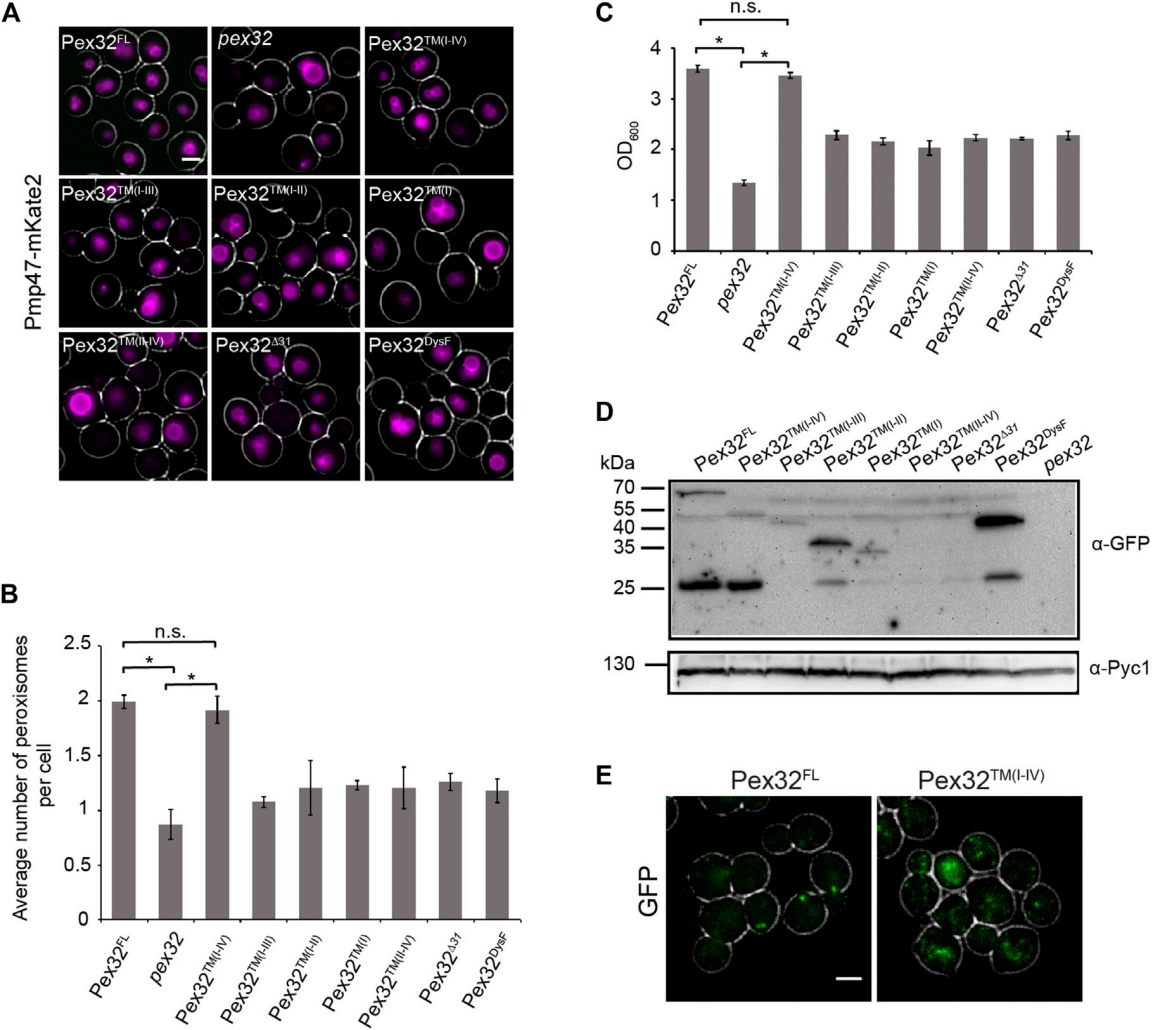
N-terminal domain of Pex32 is important for its function, while the DysF domain is required to accumulate Pex32 at peroxisome-ER contact sites. **(A)** FM analysis of methanol/glycerol-grown pex32 cells producing Pmp47-mKate2 and indicated Pex32 truncations containing GFP at the C-terminus and produced under the control of PPEX32. Scale bar: 2 μm. **(B)** Quantification of the average number of peroxisomes per cell in the indicated strains. Error bars represent s.d. from three independent experiments (*n* = 3 using 200 cells from each experiment). Significance indications: n. s. = *p* > 0.05, * = *p* < 0.01. **(C)** Optical densities of the indicated strains upon growth for 16 h on methanol/glycerol medium. Significance indications: n. s. = *p* > 0.05, * = *p* < 0.01. Error bars represent s.d. from three independent experiments. **(D)** Western blot analysis of the indicated strains. Cells were grown in a methanol/glycerol medium for 16 h. Blots were decorated with anti-GFP or anti-Pyc1 antibodies. Pyc1 was used as a loading control. **(E)** FM analysis of glucose-grown WT cells producing Pex32-GFP (left) or pex32 cells producing Pex32TM(I-IV) (right). Scale bar: 2 μm.

To analyze the role of the different domains of Pex32 in peroxisome biology, we quantified peroxisome numbers in *pex32* cells producing Pex32 truncations containing GFP at the C-terminus. To rule out overproduction artifacts, all truncations were produced under the control of the endogenous promoter (P_*PEX32*_). A WT strain producing full-length Pex32 containing GFP at the C-terminus (P_*PEX32*_Pex32^FL^-GFP) was used as a positive control, while *pex32* was included as a negative control. Peroxisomes were marked with PMP47-mKate2 and images were obtained by wide-field microscopy. Cells were grown on a mixture of glycerol and methanol to induce peroxisome proliferation. In line with our previous observations, the absence of Pex32 resulted in a strong decrease in peroxisome numbers (Figures 2A,B). Peroxisome numbers are restored to WT levels upon introduction of a construct that contains the N-terminal domain [*pex32*:Pex32™^(I−IV)^], also including first 31 N-terminal residues (Figures 2A,B). Similarly, the growth defect of *pex32* cells on glycerol/methanol was fully rescued upon introduction of the entire Pex32 N-terminal domain [*pex32*:Pex32™^(I−IV)^; Figure 2C]. None of other smaller constructs fully complemented *pex32* in terms of peroxisome numbers or growth on glycerol/methanol (Figures 2B,C). These findings indicate that the complete N-terminus (extreme N-terminal 31 residues together with the four predicted TM helices) is required and sufficient for Pex32 function. Consequently, the DysF domain is not required for the function of *H. polymorpha* Pex32 in peroxisome biology.

The western blot analysis using antibodies against GFP revealed that all Pex32 constructs were present at the expected size, except for Pex32™^(II−IV)^ and Pex32^*∆31*^, which were below the limit of detection (Figure 2D). Therefore, the inability of the latter two constructs to complement *pex32* cells maybe due to insufficient protein levels. For full-length Pex32 as well as for some of the truncations also a band of approx. 27 kDa was observed. This band most likely represents free GFP.

In glucose-grown WT cells Pex32-GFP, produced under the control of the endogenous promoter, accumulates in a single spot per cell, which represents the peroxisome-ER contact site (Wu et al., 2020) (Figure 2E, Pex32^FL^). However, upon removal of the DysF domain, GFP fluorescence was no longer concentrated in a single spot [*pex32*:Pex32™^(I−IV)^] (Figure 2E). This suggests that the DysF domain of Pex32 contributes to concentrating Pex32 at peroxisome-ER contacts.

In contrast to the DysF domain of Pex32, the DysF domain of *H. polymorpha* Pex23 is essential for its function (Supplementary Figure S2). Cells lacking Pex23 (*pex23*) or only producing the N-terminal membrane-bound domain of Pex23 without the DysF domain showed a similar phenotype as *pex23* cells (Supplementary Figure S2). Replacing the DysF domain of Pex23 with the same domain of Pex32 did not restore peroxisome formation (Supplementary Figure S2), indicating that there is no functional redundancy among the DysF domains of Pex23 and Pex32.

### The reduction of peroxisome abundance in *pex32* cells is not caused by enhanced autophagy

**FIGURE 3:**
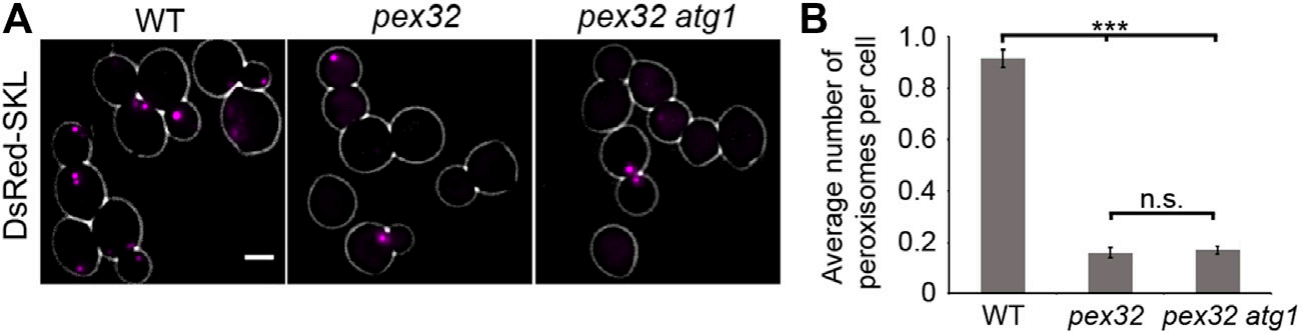
Reduced peroxisome abundance in pex32 cells is not caused by autophagy. **(A)** FM images of DsRed-SKL produced in WT, pex32, and pex32 atg1 cells grown on glucose for 4 h. Scale bar: 2 μm. **(B)** Quantification of the average number of peroxisome per cell in indicated strains. Error bars represent s.d. from three independent experiments (*n* = 3 using 200 cells from each experiment). Significance indications: n. s. = *p* > 0.05, *** = *p* < 0.001.

Deletion of *PEX32* results in a strong reduction of peroxisome numbers (Wu et al., 2020). To block autophagy, we deleted *ATG1* in *pex32*. The quantitative analysis of FM images of cells producing the peroxisomal matrix marker DsRed-SKL revealed that peroxisome numbers were similar in *pex32* and *pex32 atg1* cells (Figures 3A,B). This indicates that the reduced peroxisome numbers in *pex32* are not due to autophagy.

### Deletion of *PEX32* results in reduced Pex11 levels

**FIGURE 4:**
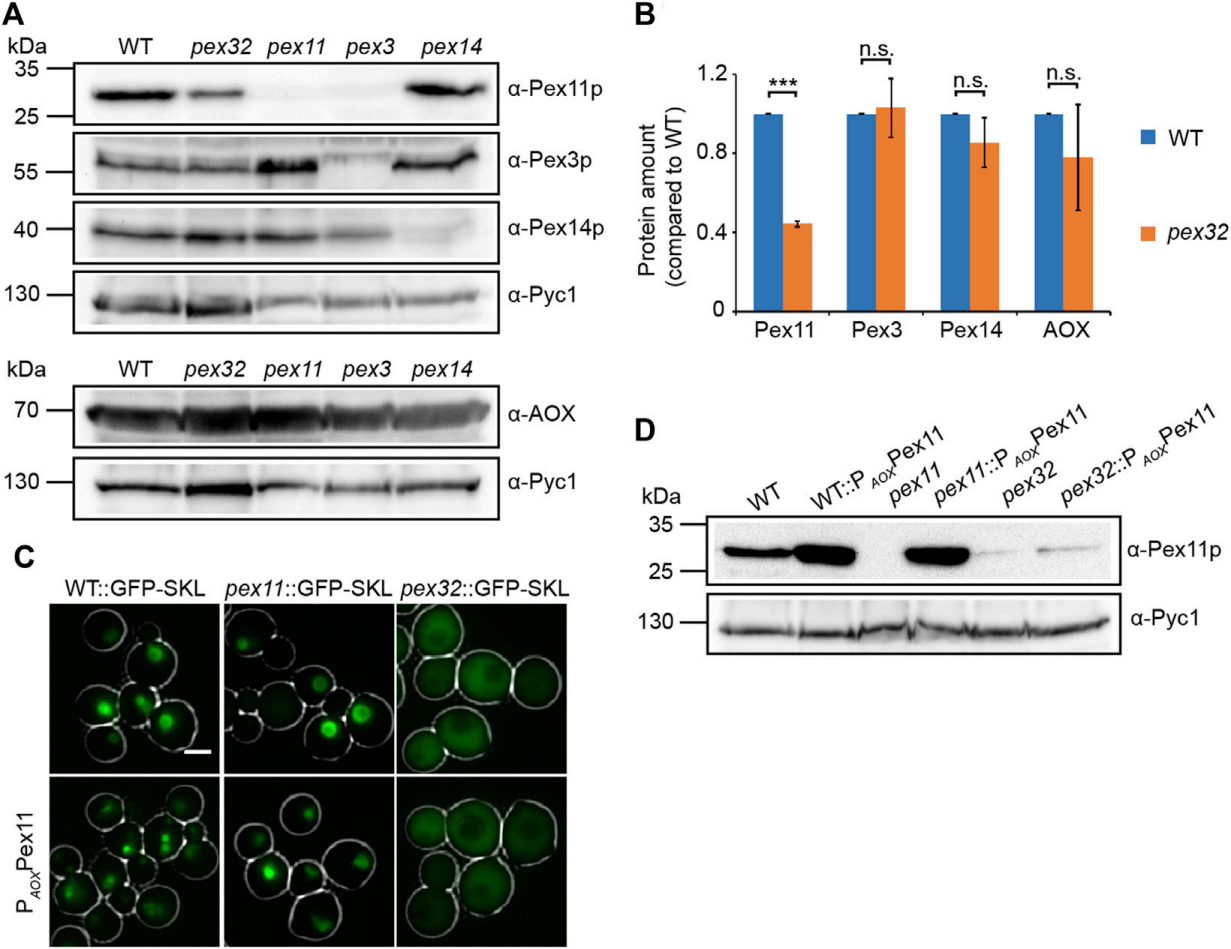
Pex11 levels are low in *pex32* cells. **(A)** Western blot analysis and **(B)** quantification of indicated proteins in WT, *pex32*, and indicated negative control cells grown for 16 h on methanol/glycerol. Blots were decorated with anti-Pex11p, anti-Pex3p, anti-Pex14p, anti-AOX, or anti-Pyc1 antibodies. Pyc1 was used as a loading control. In B, the protein levels of WT cells were set to 1. Significance indications: n. s. = *p* > 0.05, ***p = < 0.001. The error bars represent s.d*.* from three independent experiments. **(C)** FM images of WT, *pex11*, and *pex32* cells producing GFP-SKL grown on methanol/glycerol for 6 h. Scal e bar: 2 μm. **(D)** Western blot analysis of the indicated strains grown on methanol/glycerol for 6 h. Blots were decorated with anti-Pex11p or anti-Pyc1 antibodies. Pyc1 was used as a loading control.

To understand why peroxisome numbers are reduced in *pex32* cells, we analyzed the levels of several peroxisomal proteins by western blotting. As shown in Figures 4A,B the levels of the peroxisomal matrix protein alcohol oxidase (AOX) were similar in *pex32* and WT cells. Also, PMPs Pex3 and Pex14 were similar in both strains. However, Pex11 levels were strongly reduced in *pex32* cells. As reported previously, Pex11 levels are also reduced in *H. polymorpha pex3* cells, which were included as a negative control for the Pex3 blot (Knoops et al., 2014).

To investigate whether the peroxisome phenotype was restored by artificially increasing Pex11 levels, *PEX11* was overexpressed using the strong *AOX* promoter (P_*AOX*_) in *pex32* cells producing the peroxisomal matrix protein GFP-SKL. As expected, *PEX11* overexpression resulted in enhanced peroxisome proliferation in WT and *pex11* controls (Figure 4C). The western blot analysis confirmed the increase in Pex11 protein levels in these two strains. In contrast, *pex32*:P_*AOX*_ *PEX11* cells still showed mislocalization of GFP-SKL to the cytosol, like in the *pex32* control (Figure 4C). Also, Pex11 levels were not increased in *pex32* cells upon overexpression of *PEX11* (Figure 4D).

## Discussion

Here, we show that the second predicted TM in the N-terminal domain of *H. polymorpha* Pex32 is important for sorting the protein to ER, while the C-terminal DysF domain is capable to associate with peroxisomes. DysF is important to concentrate Pex32 at peroxisome-ER contact sites. Unexpectedly, the DysF domain is redundant for the function of Pex32 in peroxisome biogenesis. Finally, we show that Pex11 levels are strongly reduced in *pex32*, which explains low peroxisome abundance, as observed in *pex11* cells.

Localization studies of various truncated Pex32 variants revealed that removal of the first N-terminal 31 residues or the DysF domain had no effect on ER sorting, indicating that the region with the four predicted TM helices contains ER sorting information. The western blot analysis revealed that deletion of the first 31 N-terminal residues caused a strong decrease in protein levels (Supplementary Figure S1; Figure 2D). Possibly, the absence of this part of the protein makes Pex32 more susceptible to proteolytic degradation.

A construct consisting of only TM(I) was mainly cytosolic, while the levels of the construct consisting of TM(III-IV) were too low to allow its localization. All constructs containing TM(II) localized to ER, indicating that this domain contains ER sorting information.

We show that the DysF domain is redundant for the role of *H. polymorpha* Pex32 in peroxisome biology because peroxisome defects are restored upon introduction of a Pex32 construct lacking the DysF domain in *pex32* cells. This observation differs from earlier findings in *P. pastoris* and *S. cerevisiae*. In *P. pastoris*, DysF domains of Pex30 and Pex31 are essential for the regulation of peroxisome number and size (Yan et al., 2008). Similarly, the DysF domain of *S. cerevisiae* Pex30 was shown to be essential for normal peroxisome biology (Ferreira and Carvalho, 2021). Moreover, removal of the ScPex30 DysF domain results in defects in the NVJ organization and lipid body clustering (Ferreira and Carvalho, 2021).

Our data show that accumulation of HpPex32-GFP at peroxisome-ER contact sites requires the DysF domain because GFP fluorescence no longer is present in a single spot per cell when the DysF motif was removed. Moreover, we showed that the Pex32 DysF domain has the ability to associate with peroxisomes. Most likely the Pex32 DysF domain has a binding partner at the peroxisomal membrane, which keeps the protein concentrated at peroxisome-ER contact sites. This accumulation apparently is not essential, because a construct lacking the DysF domain, which does not accumulate at contact sites, still can functionally complement a *pex32* deletion strain. Possibly in these cells, sufficient Pex32 is present at the contact sites for their function.

We show that cells lacking Pex32 have strongly reduced Pex11 levels, while the levels of other peroxisomal proteins tested were normal. Moreover, upon placing *PEX11* under the control of a strong promoter in *pex32* cells, Pex11 protein levels were not enhanced, suggesting that Pex32 is required to maintain normal Pex11 levels. This observation may explain why in *pex32* cells peroxisome numbers are strongly reduced as observed for *pex11* cells. Why Pex11 levels are low in *pex32* is not yet understood and requires further analysis.

## Materials and methods

### Strains and growth conditions

*H*. *polymorpha* cells were grown in batch cultures at 37°C on mineral media (Van Dijken et al., 1976) supplemented with 0.5% glucose or 0.5% methanol, or a mixture of 0.5% methanol and 0.05% glycerol as carbon source. When required leucine was added to a final concentration of 60 μg/ml. For growth on plates, YPD media (1% yeast extract, 1% peptone, and 1% glucose) or YND media (0.67% yeast nitrogen base without amino acids (YNB; Difco; BD) and 0.5% glucose) were supplemented with 2% agar. Resistant transformants were selected using 100 μg/ml zeocin (Invitrogen), 100 μg/ml nourseothricin (WERNER BioAgents), or 300 μg/ml hygromycin (Invitrogen).

*Escherichia coli* strain DH5α was used for cloning. *E*. *coli* cells were grown at 37°C in Luria broth (LB) media (1% Bactotryptone, 0.5% Yeast Extract, and 0.5% NaCl) supplemented with 100 μg/ml ampicillin or 50 μg/ml kanamycin. 2% agar was added in LB medium for growth on plates.

### Construction of *H*. *polymorpha* strains

All strains, plasmids, and primers used in this study are listed in Supplementary Tables S1, S2, and S3, respectively. Plasmid integration was performed as described previously (Faber et al., 1994). All integrations were confirmed by PCR. Gene deletions were confirmed by PCR and Southern blotting.

### Construction of strains expressing GFP-tagged Pex32 truncations

Plasmids encoding P_*ADH1*_Pex32™^(I−IV)^-mGFP and P_*ADH1*_Pex32^DysF^-mGFP were constructed as follows: a PCR fragment encoding the N-terminus (all 4 TMs) of *PEX32* was obtained using primers Fw Pex32_1-696_ and Rv Pex32_1-696_ with *H*. *polymorpha yku80* genomic DNA as a template. Similarly, a PCR fragment encoding the DysF domain of *PEX32* was obtained with primers Fw Pex32_697-1062_ and Rv Pex32_697-1062_. The obtained PCR fragments were digested with *Hin*dIII and *Bgl*II and separately inserted between the *Hin*dIII and *Bgl*II sites of plasmid pHIPZ18-*INP1*-GFP, resulting in pHIPZ18-*PEX32*™^(I−IV)^-mGFP and pHIPZ18-*PEX32* ^DysF^-mGFP. Both plasmids and pAMK106 were digested by *Hin*dIII and *Sal*I separately, and ligated, resulting in pHIPN18-*PEX32*™^(I−IV)^-mGFP and pHIPN18-*PEX32* ^DysF^-mGFP.

By using pHIPN18-*PEX32*™^(I−IV)^-mGFP as the template, primer pairs: 1) Fw Pex32_1-696_/Rev Pex32_1-501_, 2) Fw Pex32_1-696_/Rev Pex32_1-312_, and 3) Fw Pex32_1-696_/Rev Pex32_1-177_ were used to amplify constructs containing: 1) *PEX32*™^(I−III)^, 2) *PEX32*™^(I−II)^, and 3) *PEX32*™^(I)^, respectively. PCR products were digested with *Hin*dIII and *Bgl*II, and inserted between the *Hin*dIII and *Bgl*II sites of pHIPN18-*PEX32*™^(I−IV)^-mGFP separately to obtain pHIPN18-*PEX32*™^(I−III)^-mGFP, pHIPN18-*PEX32*™^(I−II)^-mGFP, and pHIPN18-*PEX32*™^(I)^-mGFP.

Plasmids pHIPN18-*PEX32*™^(II−IV)^-mGFP and pHIPN18-*PEX32* ^∆31^-mGFP were constructed by using the same method. *H*. *polymorpha* Pex32-mGFP genomic DNA was used as the template, using primer pairs Fw Pex32_(169–1062)_/Rv DysF_*PEX32*-mGFP_ and Fw Pex32_(94–1062)_/Rv DysF_*PEX32*-mGFP_ to amplify fragments containing *PEX32*™^(II−IV)^-mGFP and *PEX32* ^∆31^-mGFP, respectively. Both PCR products and pHIPN18-*PEX32*™^(I−IV)^-GFP were restricted by *Hin*dIII and *Xho*I separately, resulting in pHIPN18-*PEX32*™^(II−IV)^-mGFP and pHIPN18-*PEX32* ^∆31^-mGFP.

The plasmid for *PEX32* overexpression was constructed as follows: a PCR fragment containing full-length *PEX32* was obtained using primers Fw Pex32_1-696_ and Rv DysF_PEX32-mGFP_ with Pex32-mGFP strain as a template. The PCR product and pHIPN18-Pex32™^(I−IV)^-mGFP were digested by *Hin*dIII and *Xho*I and ligated resulting in pHIPN18-*PEX32*-mGFP.

All aforementioned plasmids were linearized with *Aat*II and integrated into *pex32* strains separately to produce P_*ADH1*_Pex32-mGFP, P_*ADH1*_Pex32™^(I−IV)^-mGFP, P_*ADH1*_Pex32™^(I−III)^-mGFP, P_*ADH1*_Pex32™^(I−II)^-mGFP, P_*ADH1*_Pex32™^(I)^-mGFP, P_*ADH1*_Pex32™^(II−IV)^-mGFP, P_*ADH1*_Pex32^∆31^-mGFP, or P_*ADH1*_Pex32^DysF^-mGFP. *Dra*I-linearized pHIPX7-BiP_*N30*_ *-*mCherry-HDEL was integrated into various truncations independently to express BiP-mCherry-HDEL.

To obtain pHIPN18-*PEX32*™^(II)^-mGFP and pHIPN18-*PEX32*™^(III−IV)^-mGFP, plasmid pHIPN18-*PEX32*™^(I−IV)^-mGFP was used as a template, and primer pairs Fw Pex32_o2TM_/Rev Pex32_1-312_, Fw Pex32_TM3+4_/Rv Pex32_1-696_ were used to amplify fragments containing *PEX32*™^(II)^ and *PEX32*™^(II−IV)^, respectively. These PCR products and pHIPN18-*PEX32*™^(I−IV)^-mGFP were digested with *Hin*dIII and *Bgl*II and ligated to obtain pHIPN18-*PEX32*™^(II)^-mGFP and pHIPN18-*PEX32*™^(III−IV)^-mGFP. *Aat*II-linearized plasmids were integrated into *pex32*:BiP-mCherry-HDEL separately to produce P_*ADH1*_Pex32™^(II)^-mGFP and P_*ADH1*_Pex32™^(III−IV)^-mGFP.

Plasmids for producing various Pex32 truncations under the control of the *PEX32* promoter were constructed as follows: PCR was performed on *yku80* genomic DNA to amplify the *PEX32* promoter using primers P_*PEX32*_ fw and P_*PEX32*_ rev. The obtained PCR fragment was digested with *Not*I and *Hin*dIII, and then replaced the *ADH1* promoter (P_*ADH1*_) in *Not*I/*Hin*dIII digested variants of Pex32 truncations. All constructions under the control of P_*PEX32*_ were linearized with *Eco*RV and integrated into *pex32*:Pmp47-mKate2 cells separately to produce Pex32™^(I−IV)^-mGFP, Pex32™^(I−III)^-mGFP, Pex32™^(I−II)^-mGFP, Pex32™^(I)^-mGFP, Pex32™^(II−IV)^-mGFP, Pex32^∆31^-mGFP, and Pex32^DysF^-mGFP.

### Construction of the *pex32 atg1* double deletion strain

To construct *pex32 atg1*, a PCR fragment containing the *ATG1* deletion cassette was amplified with primers pDEL-ATG1-fwd and pDEL-ATG1-rev using plasmid pARM011 as a template. The resulting *ATG1* deletion cassette was transformed into *pex32* cells to get a double mutant of *pex32 atg1*. *Dra*I-linearized pAMK15 plasmid was transformed into *pex32 atg1* cells to produce DsRed-SKL.

### Construction of strains expressing *PEX11* under control of the alcohol oxidase promoter (P_*AOX*_)

Plasmid pHIPH4-*PEX11* was produced by ligation of *Not*I and *Sma*I digested pHIPX4-*PEX11* and pHIPH7-*PEX11*. The plasmid pHIPX4-*PEX11* was constructed as follows: a PCR fragment containing *PEX11* was obtained using primers Pex11-3 and Pex11-4 with WT genomic DNA as a template. The PCR product and pHIPX4 were restricted by *Hin*dIII and *Sal*I, ligated which result in pHIPX4-*PEX11*. pHIPH7-*PEX11* was constructed from the ligation of *Bam*HI and *Xma*I digested pHIPH5-*PEX11* and pHIPH7-DsRed-SKL. To get pHIPH5-*PEX11*, the *PEX11* gene was amplified with primers PEX11-01 and PEX11-02 by using the WT genomic DNA as templates, *Bam*HI and *Xma*I digested PCR fragment was inserted between *Bam*HI and *Xma*I sites of pSEM04. *Nsi*I-linearized pHIPH4-*PEX11* was integrated into WT:GFP-SKL, *pex11*:GFP-SKL, and *pex32*:GFP-SKL strains, respectively, to produce P_*AOX*_Pex11.

### Preparation of yeast TCA lysates, SDS-PAGE, and western blotting

Cell extracts of TCA-treated cells were prepared for SDS-PAGE as described previously (Baerends et al., 2000). Equal amounts of protein were loaded per lane and blots were probed with anti-mGFP antibodies (sc-9996, Santa Cruz Biotech; 1:2000 dilution), anti-Pex11 antibodies (Knoops et al., 2014; 1:2,000 dilution), anti-Pex14 antibodies (Komori et al., 1997; 1:10,000 dilution), anti-Pex3 antibodies (Baerends et al., 1997; 1:5,000 dilution), anti-AOX antibodies (van der Klei et al., 1995; 1:10,000 dilution), or anti-pyruvate carboxylase 1 (Pyc1) antibodies (Ozimek et al., 2007; 1:10,000 dilution). Secondary goat anti-rabbit (31,460) or goat anti-mouse (31,430) antibodies conjugated to horseradish peroxidase (HRP) (Thermo Scientific; 1:5,000 dilution) were used for detection. Pyc1 was used as a loading control.

### Quantification of western blots

Blots were scanned using a densitometer (Bio-Rad, GS-710) and protein levels were quantified using ImageJ software. The intensity of each band measured was normalized by dividing by the intensity of the corresponding Pyc1 band (loading control). Normalized values obtained for Pex11, Pex3, Pex14, and AOX levels in WT cells were set to one and levels in *pex32* cells were displayed relative to WT control. Standard deviations were calculated using Excel. Significance was determined using two-tailed Student’s *t*-test. n. s. represents *p*-values > 0.05 and *** represents *p*-values < 0.001. The data presented are derived from three independent experiments.

### Fluorescence microscopy

Wide-field FM images were captured at room temperature using a 100 × 1.30 NA objective (Carl Zeiss, Oberkochen, Germany). Images were acquired using a Zeiss Axioscope A1 fluorescence microscope (Carl Zeiss), Micro-Manager 1.4 software, and a CoolSNAP HQ^2^ digital camera. GFP fluorescence was visualized with a 470/40 nm band-pass excitation filter, a 495 nm dichromatic mirror, and a 525/50 nm band-pass emission filter. DsRed fluorescence was visualized with a 546/12 nm band-pass excitation filter, a 560 nm dichromatic mirror, and a 575–640 nm band-pass emission filter. mCherry and mKate2 fluorescence were visualized with a 587/25 nm band-pass excitation filter, a 605 nm dichromatic mirror, and a 670/70 nm band-pass emission filter.

Airy-scan images were captured with a confocal microscope (LSM800; Carl Zeiss) equipped with a 32-channel gallium arsenide phosphide photomultiplier tube (GaAsP-PMT), Zen 2009 software (Carl Zeiss) and a 63 × 1.40 NA objective (Carl Zeiss, Oberkochen, Germany). The GFP, mKate2, and mCherry fluorescence were visualized with a 488, 561, and 587 nm laser, respectively.

Image analysis was performed using ImageJ. Bright field images have been adjusted to only show cell outlines. Figures were prepared using Adobe Illustrator software.

### Quantification of peroxisomes numbers

Peroxisome numbers were quantified using 200 randomly selected cells from three independent cultures. Numbers correspond to the average number of peroxisomes per cell. Standard deviations were calculated using Excel. Significance was determined using two-tailed Student’s *t*-test. n. s. represents *p*-values > 0.05 and *** represents *p*-values < 0.001.

## Data Availability

The raw data supporting the conclusion of this article will be made available by the authors, without undue reservation.

## Supplementary material

The Supplementary Material for this article can be foundSupplementary Material online at: https://www.frontiersin.org/articles/10.3389/fcell.2022.957871/full#supplementary-material
