# Supplementary material for: Structure–function analysis of the ER-peroxisome contact site protein Pex32
Source: Front Cell Dev Biol. 2022 Aug 9;10:957871. doi: 10.3389/fcell.2022.957871 (PMC9395739; doi:10.3389/fcell.2022.957871)
Supplement: Supplementary file 1 [file DataSheet1.PDF]

Supplementary Material.

Supplementary Figures

Supplementary figure S1

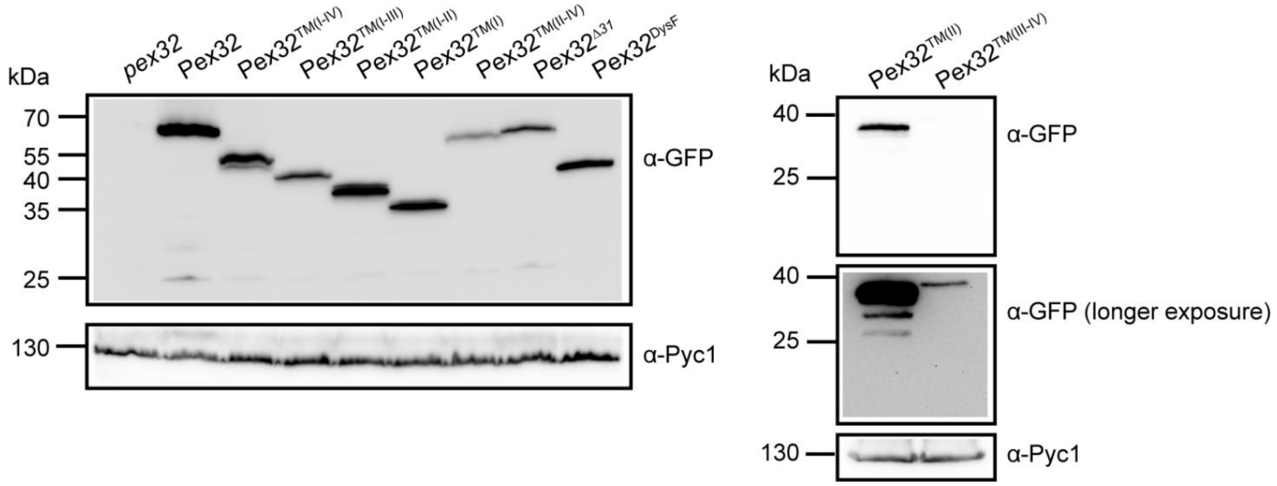

Supplementary Figure S1: Western blot analysis of the indicated strains.

Western blot analysis of the indicated strains grown on glucose for 4 h. Blots were decorated with anti-GFP or anti-Pyc1 antibodies. Pyc1 was used as a loading control.

Supplementary figure S2

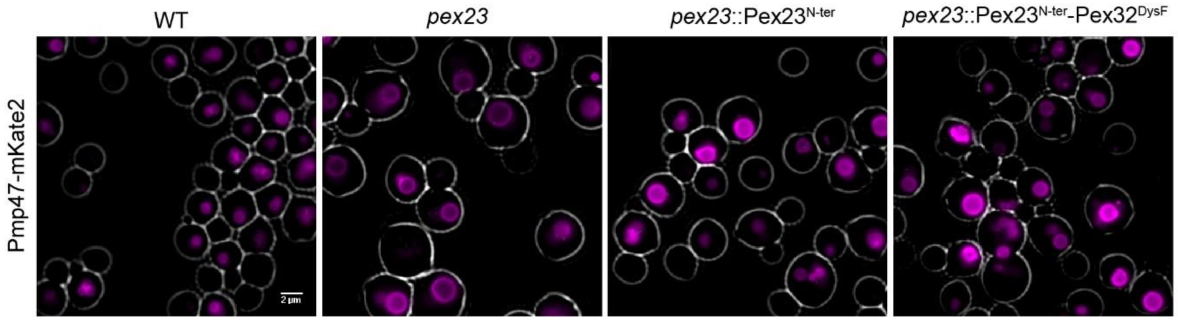

Supplementary Figure S2: Replacing the DysF domain of Pex23 by the DysF domain of Pex32 does not result in a functional protein for peroxisome biogenesis. Fluorescence microscopy images of cells of the indicated strains producing Pmp47-mKate2. Cells were grown on methanol for 16 h. Scale bar: 2 µm.

The absence of Pex23 (*pex23*) results in less and larger peroxisomes. A similar phenotype is observed when the N-terminal domain of Pex23 (Pex23$^{N\text{-}ter}$) is re-introduced in *pex23* cells or when the DysF domain of Pex32 was added to the Pex23 N-terminus (*pex23*::Pex23$^{N\text{-}ter}$-Pex32^{DysF}).

Strain construction

A plasmid encoding pHIPN23-*PEX23*$^{N\text{-}ter}$-mGFP was constructed as follows: the Pex23 N-terminus (until the DysF domain) was amplified by PCR using primers Fw- or Rv-Nter-Pex23 and using genomic DNA of strain *yku80* as a template. The obtained fragment and plasmid pHIPN-*PEX14*-mGFP were digested with *Hin*dIII and *Bgl*II, ligated resulting in plasmid pHIPN-*PEX23*$^{N\text{-}ter}$-mGFP. *H. polymorpha* Pex23 endogenous promoter was amplified by using primers Fw- or Rv-P-Pex23 and using *yku80* genomic DNA as a template. The obtained PCR fragment and plasmid pHIPN-*PEX23*$^{N\text{-}ter}$-mGFP were restricted with *Bam*HI and *Hin*dIII, ligated resulting in plasmid pHIPN23-*PEX23*$^{N\text{-}ter}$-mGFP. *Nde*I linearized plasmid was integrated into *pex23* cells to produce Pex23$^{N\text{-}ter}$-mGFP. Subsequently, *Spe*I-linearized pHIPX-*PMP47*-mKate2 was integrated into *pex23*::Pex23$^{N\text{-}ter}$-mGFP cells to produce Pmp47-mKate2.

To fuse Pex32 DysF, plasmid pHIPH5-*PEX32*-mGFP was used as a template to amplify the Pex32^{DysF}-mGFP fragment with primers Fw-Cter-Pex32 and Rv-GFP-Pex32. The PCR product and plasmid pHIPN23-*PEX23*$^{N\text{-}ter}$-mGFP were restricted with *Bgl*II and *Nsi*I, ligated resulting in plasmid pHIPN23-*PEX23*$^{N\text{-}ter}$-*PEX32*^{DysF}-mGFP. This plasmid was linearized with *Nde*I and integrated into *pex23* cells to produce Pex23$^{N\text{-}ter}$-Pex32^{DysF}-mGFP. *Spe*I-linearized pHIPX-*PMP47*-mKate2 was integrated into *pex23*::Pex23$^{N\text{-}ter}$-Pex32^{DysF}-mGFP cells to produce Pmp47-mKate2.

Supplementary Table 1: Strains used in this study

Strain	Description	Reference
WT	NCYC495; *leu 1.1*	(Sudbery et al., 1988)
WT:: *yku80*	NCYC495 *yku80* deletion strain; *leu 1.1*, *URA3*	(Saraya et al., 2012)
pex32	*yku80* with *PEX32* deletion strain; *leu 1.1*, *URA3*, Zeo^R	(Wu et al., 2020)
Pex32-mGFP	*yku80* with pHIPZ-*PEX32*-mGFP; *leu 1.1*, *URA3*, Zeo^R	(Wu et al., 2020)
pex32::P_{ADH1}Pex32-mGFP	*pex32* with pHIPN18-*PEX32*-mGFP; *leu 1.1*, *URA3*, Zeo^R, Nat^R	This study
pex32::$P_{ADH1}$$Pex32^{TM(I-IV)}$-mGFP	*pex32* with pHIPN18-$PEX32^{TM(I-IV)}$-mGFP; *leu 1.1*, *URA3*, Zeo^R, Nat^R	This study
pex32::$P_{ADH1}$$Pex32^{TM(I-III)}$-mGFP	*pex32* with pHIPN18-$PEX32^{TM(I-III)}$-mGFP; *leu 1.1*, *URA3*, Zeo^R, Nat^R	This study
pex32::$P_{ADH1}$$Pex32^{TM(I-II)}$-mGFP	*pex32* with pHIPN18-$PEX32^{TM(I-II)}$-mGFP; *leu 1.1*, *URA3*, Zeo^R, Nat^R	This study
pex32::$P_{ADH1}$$Pex32^{TM(I)}$-mGFP	*pex32* with pHIPN18-$PEX32^{TM(I)}$-mGFP; *leu 1.1*, *URA3*, Zeo^R, Nat^R	This study
pex32::$P_{ADH1}$$Pex32^{TM(II-IV)}$-mGFP	*pex32* with pHIPN18-$PEX32^{TM(II-IV)}$-mGFP; *leu 1.1*, *URA3*, Zeo^R, Nat^R	This study
pex32::$P_{ADH1}$$Pex32^{\Delta 31}$-mGFP	*pex32* with PHIPN18-$PEX32^{\Delta 31}$-mGFP; *leu 1.1*, *URA3*, Zeo^R, Nat^R	This study
pex32::$P_{ADH1}$$Pex32^{DysF}$-mGFP	*pex32* with PHIPN18-$PEX32^{DysF}$-mGFP; *leu 1.1*, *URA3*, Zeo^R, Nat^R	This study
pex32::BiP-mCherry-HDEL	*pex32* with pHIPX7-BiP_{N30}-mCherry-HDEL; *URA3*, Zeo^R, *LEU2*	This study
pex32::P_{ADH1}Pex32-mGFP::BiP-mCherry-HDEL	*pex32*::P_{ADH1}Pex32-mGFP with pHIPX7-BiP_{N30}-mCherry-HDEL; *URA3*, Zeo^R, Nat^R, *LEU2*	This study
pex32::$P_{ADH1}$$Pex32^{TM(I-IV)}$-mGFP::BiP-mCherry-HDEL	*pex32*::$P_{ADH1}$$Pex32^{TM(I-IV)}$-mGFP with pHIPX7-$BiP_{N30}$-mCherry-HDEL; *URA3*, Zeo^R, Nat^R, *LEU2*	This study
pex32::$P_{ADH1}$$Pex32^{TM(I-III)}$-mGFP::Bip-mCherry-HDEL	*pex32*::$P_{ADH1}$$Pex32^{TM(I-III)}$-mGFP with pHIPX7-$BiP_{N30}$-mCherry-HDEL; *URA3*, Zeo^R, Nat^R, *LEU2*	This study
pex32::$P_{ADH1}$$Pex32^{TM(I-II)}$-mGFP::BiP-mCherry-HDEL	*pex32*::$P_{ADH1}$$Pex32^{TM(I-II)}$-mGFP with pHIPX7-$BiP_{N30}$-mCherry-HDEL; *URA3*, Zeo^R, Nat^R, *LEU2*	This study
pex32::$P_{ADH1}$$Pex32^{TM(I)}$-mGFP::BiP-mCherry-HDEL	*pex32*::$P_{ADH1}$$Pex32^{TM(I)}$-mGFP with pHIPX7-$BiP_{N30}$-mCherry-HDEL; *URA3*, Zeo^R, Nat^R, *LEU2*	This study
pex32::$P_{ADH1}$$Pex32^{TM(II-IV)}$-	*pex32*::$P_{ADH1}$$Pex32^{TM(II-IV)}$-mGFP with pHIPX7-$BiP_{N30}$-	This study

mGFP::BiP-mCherry-HDEL	mCherry-HDEL; *URA3*, Zeo^{R}, Nat^{R}, *LEU2*	
pex32::P$_{ADH1}$Pex32$^{\Delta 31}$-mGFP::BiP-mCherry-HDEL	*pex32*::P$_{ADH1}$Pex32$^{\Delta 31}$-mGFP with pHIPX7-BiP$_{N30}$-mCherry-HDEL; *URA3*, Zeo^{R}, Nat^{R}, *LEU2*	This study
pex32::P$_{ADH1}$Pex32^{DysF}-mGFP::BiP-mCherry-HDEL	*pex32*::P$_{ADH1}$Pex32^{DysF}-mGFP with pHIPX7-BiP$_{N30}$-mCherry-HDEL; *URA3*, Zeo^{R}, Nat^{R}, *LEU2*	This study
pex32::P$_{ADH1}$Pex32$^{TM(II)}$-mGFP::BiP-mCherry-HDEL	*pex32::BiP-mCherry-HDEL* with pHIPN18-*PEX32*$^{TM(II)}$-mGFP; *URA3*, Zeo^{R}, *LEU2*, Nat^{R}	This study
pex32::P$_{ADH1}$Pex32$^{TM(III-IV)}$-mGFP::BiP-mCherry-HDEL	*pex32::BiP-mCherry-HDEL* with pHIPN18-*PEX32*$^{TM(III-IV)}$-mGFP; *URA3*, Zeo^{R}, *LEU2*, Nat^{R}	This study
pex32::Pmp47-mKate2	*pex32* with pHIPX-*PMP47*-mKate2*;* *URA3*, Zeo^{R}, *LEU2*	This study
Pex32^{FL}-mGFP::Pmp47-mKate2	Pex32-mGFP with pHIPX-*PMP47*-mKate2*;* *URA3*, Zeo^{R}, *LEU2*	This study
pex32::Pex32$^{TM(I-IV)}$-mGFP::Pmp47-mKate2	*pex32*::Pmp47-mKate2 with pHIPN22-*PEX32*$^{TM(I-IV)}$-mGFP; *URA3*, Zeo^{R}, *LEU2*, Nat^{R}	This study
pex32::Pex32$^{TM(I-III)}$-mGFP::Pmp47-mKate2	*pex32*::Pmp47-mKate2 with pHIPN22-*PEX32*$^{TM(I-III)}$-mGFP; *URA3*, Zeo^{R}, *LEU2*, Nat^{R}	This study
pex32::Pex32$^{TM(I-II)}$-mGFP::Pmp47-mKate2	*pex32*::Pmp47-mKate2 with pHIPN22-*PEX32*$^{TM(I-II)}$-mGFP; *URA3*, Zeo^{R}, *LEU2*, Nat^{R}	This study
pex32::Pex32$^{TM(I)}$-mGFP::Pmp47-mKate2	*pex32*::Pmp47-mKate2 with pHIPN22-*PEX32*$^{TM(I)}$-mGFP; *URA3*, Zeo^{R}, *LEU2*, Nat^{R}	This study
pex32::Pex32$^{TM(II-IV)}$-mGFP::Pmp47-mKate2	*pex32*::Pmp47-mKate2 with pHIPN22-*PEX32*$^{TM(II-IV)}$-mGFP; *URA3*, Zeo^{R}, *LEU2*, Nat^{R}	This study
pex32::Pex32$^{\Delta 31}$-mGFP::Pmp47-mKate2	*pex32*::Pmp47-mKate2 with pHIPN22-*PEX32*$^{\Delta 31}$-mGFP; *URA3*, Zeo^{R}, *LEU2*, Nat^{R}	This study
pex32::Pex32^{DysF}-mGFP::Pmp47-mKate2	*pex32*::Pmp47-mKate2 with pHIPN22-*PEX32*^{DysF}-mGFP; *URA3*, Zeo^{R}, *LEU2*, Nat^{R}	This study
pex23	*yku80* with *PEX23* deletion strain; *leu 1.1*, *URA3*, Zeo^{R}	(Wu et al., 2020)
pex23::Pex23^{N-ter}-mGFP	*pex23* with pHIPN23-*PEX23*$^{N-ter}$-mGFP; *leu 1.1*, *URA3*, Zeo^{R}, Nat^{R}	This study
pex23::Pex23^{N-ter}-mGFP::Pmp47-mKate2	*pex23*::Pex23^{N-ter}-mGFP with pHIPX-*PMP47*-mKate2*;* *URA3*, Zeo^{R}, Nat^{R}, *LEU2*	This study
pex23::Pex23^{N-ter}-Pex32^{DysF}-mGFP	*pex23* with pHIPN23-*PEX23*$^{N-ter}$-*PEX32*^{DysF}-mGFP; *leu 1.1*, *URA3*, Zeo^{R}, Nat^{R}	This study
pex23::Pex23^{N-ter}-Pex32^{DysF}-mGFP::Pmp47-mKate2	*pex23*::Pex23^{N-ter}-Pex32^{DysF}-mGFP with pHIPX-*PMP47*-mKate2*;* *URA3*, Zeo^{R}, Nat^{R}, *LEU2*	This study

pex32 atg1	*pex32* with *ATG1* deletion cassette; *leu 1.1*, *URA3*, Zeo^R, Hph^R	This study
pex32 atg1::DsRed-SKL	*pex32 atg1* with pAMK15; *URA3*, Zeo^R, Hph^R, *LEU2*	This study
WT::DsRed-SKL	*yku80* with pHIPN18-DsRed-SKL; *leu 1.1*, *URA3*, Nat^R	(Wu et al., 2020)
pex32::DsRed-SKL	*pex32* with pAMK15; *URA3*, Zeo^R, *LEU2*	(Wu et al., 2020)
pex11	*PEX11* deletion strain; *leu 1.1*, *URA3*	(Krikken et al., 2009)
pex3	*PEX3* deletion strain; *leu 1.1*, *URA3*	(Baerends et al., 1997)
pex14	*PEX14* deletion strain; *leu 1.1*, *URA3*	(Komori et al., 1997)
WT::GFP-SKL	*yku80* with pFEM35; *URA3*, *LEU2*	(Krikken et al., 2009)
WT::GFP-SKL::P_{AOX}Pex11	WT::GFP-SKL with pHIPH4-*PEX11*; *URA3*, *LEU2*, Hph^R	This study
pex11::GFP-SKL	*pex11* with pFEM35; *URA3*, *LEU2*	(Krikken et al., 2009)
pex11::GFP-SKL::P_{AOX}Pex11	*pex11*::GFP-SKL with pHIPH4-*PEX11*; *URA3*, *LEU2*, Hph^R	This study
pex32::GFP-SKL	*pex32* with pFEM35; *URA3*, Zeo^R, *LEU2*	(Wu et al., 2020)
pex32::GFP-SKL::P_{AOX}Pex11	*pex32*::GFP-SKL with pHIPH4-*PEX11*; *URA3*, *LEU2*, Hph^R	This study

Supplementary Table 2: Plasmids used in this study

Plasmid	Description	Reference
pHIPZ18-*INP1*-GFP	pHIPZ plasmid containing the full length of *INP1* fused with mGFP under the control of *ADH1* promoter; Zeo^R, Amp^R	(Krikken et al., 2020)
pHIPZ18-*PEX32*$^{TM(I-IV)}$-mGFP	pHIPZ plasmid containing *PEX32*$^{TM(I-IV)}$ fused with mGFP under the control of *ADH1* promoter; Zeo^R, Amp^R	This study
pHIPZ18-*PEX32*^{DysF}-mGFP	pHIPZ plasmid containing *PEX32*^{DysF} fused with mGFP under the control of *ADH1* promoter; Zeo^R, Amp^R	This study
pAMK106	pHIPN plasmid containing eGFP-SKL under the control of *ADH1* promoter; Nat^R, Amp^R	(Krikken et al., 2020)
pHIPN18-Pex32$^{TM(I-IV)}$-mGFP	pHIPN plasmid containing *PEX32*$^{TM(I-IV)}$ fused with mGFP under the control of *ADH1* promoter; Nat^R, Amp^R	This study
pHIPN18-*Pex32*^{DysF}-mGFP	pHIPN plasmid containing *PEX32*^{DysF} fused with mGFP under the control of *ADH1* promoter; Nat^R, Amp^R	This study
pHIPN18-*PEX32*$^{TM(I-III)}$-mGFP	pHIPN plasmid containing *PEX32*$^{TM(I-III)}$ fused with mGFP under the control of *ADH1* promoter; Nat^R, Amp^R	This study
pHIPN18-*PEX32*$^{TM(I-II)}$-mGFP	pHIPN plasmid containing *PEX32*$^{TM(I-II)}$ fused with mGFP under the control of *ADH1* promoter; Nat^R, Amp^R	This study
pHIPN18-*PEX32*$^{TM(I)}$-mGFP	pHIPN plasmid containing *PEX32*$^{TM(I)}$ fused with mGFP under the control of *ADH1* promoter; Nat^R, Amp^R	This study
pHIPN18-*PEX32*$^{TM(II-IV)}$-mGFP	pHIPN plasmid containing *PEX32*$^{TM(II-IV)}$ fused with mGFP under the control of *ADH1* promoter; Nat^R, Amp^R	This study
pHIPN18-*PEX32*$^{\Delta 31}$-mGFP	pHIPN plasmid containing *PEX32*$^{\Delta 31}$ fused with mGFP under the control of *ADH1* promoter; Nat^R, Amp^R	This study
pHIPN18-*PEX32*-mGFP	pHIPN plasmid containing the full length of *PEX32* fused with GFP under the control of *ADH1* promoter; Nat^R, Amp^R	This study
pHIPX7-BiP$_{N30}$-mCherry-HDEL	pHIPX plasmid containing BiP$_{N30}$ fused to mCherry-HDEL under control of *TEF* promoter; *LEU2*, Kan^R	(Wu et al., 2020)
pHIPX-*PMP47*-mKate2	pHIPX plasmid containing the C-terminal region of *PMP47* fused with mKate2; *LEU2,* Amp^R	(Krikken et al., 2020)
pHIPN18-*PEX32*$^{TM(II)}$-mGFP	pHIPN plasmid containing *PEX32*$^{TM(II)}$ fused with	This study

	mGFP under the control of *ADH1* promoter; Nat^{R}, Amp^{R}	
pHIPN18-*PEX32*$^{TM(III\text{-}IV)}$-mGFP	pHIPN plasmid containing *PEX32*$^{TM(III\text{-}IV)}$ fused with mGFP under the control of *ADH1* promoter; Nat^{R}, Amp^{R}	This study
pHIPN22-*PEX32*$^{TM(I\text{-}IV)}$-mGFP	pHIPN plasmid containing *PEX32*$^{TM(I\text{-}IV)}$ fused with mGFP under the control of *PEX32* promoter; Nat^{R}, Amp^{R}	This study
pHIPN22-*PEX32*$^{TM(I\text{-}III)}$-mGFP	pHIPN plasmid containing *PEX32*$^{TM(I\text{-}III)}$ fused with mGFP under the control of *PEX32* promoter; Nat^{R}, Amp^{R}	This study
pHIPN22-*PEX32*$^{TM(I\text{-}II)}$-mGFP	pHIPN plasmid containing *PEX32*$^{TM(I\text{-}II)}$ fused with mGFP under the control of *PEX32* promoter; Nat^{R}, Amp^{R}	This study
pHIPN22-*PEX32*$^{TM(I)}$-mGFP	pHIPN plasmid containing *PEX32*$^{TM(I)}$ fused with mGFP under the control of *PEX32* promoter; Nat^{R}, Amp^{R}	This study
pHIPN22-*PEX32*$^{TM(II\text{-}IV)}$-mGFP	pHIPN plasmid containing *PEX32*$^{TM(II\text{-}IV)}$ fused with mGFP under the control of *PEX32* promoter; Nat^{R}, Amp^{R}	This study
pHIPN22-*PEX32*$^{\Delta 31}$-mGFP	pHIPN plasmid containing *PEX32*$^{\Delta 31}$ fused with mGFP under the control of *PEX32* promoter; Nat^{R}, Amp^{R}	This study
pHIPN22-*PEX32*^{DysF}-mGFP	pHIPN plasmid containing *PEX32*^{DysF} fused with mGFP under the control of *PEX32* promoter; Nat^{R}, Amp^{R}	This study
pHIPN-*PEX14*-mGFP	pHIPN plasmid containing C-terminal region of *PEX14* fused with mGFP; Nat^{R}, Amp^{R}	(Wu et al., 2020)
pHIPN-*PEX23*$^{N\text{-}ter}$-mGFP	pHIPN plasmid containing N-terminal region of *PEX23* fused with mGFP; Nat^{R}, Amp^{R}	This study
pHIPN23-*PEX23*$^{N\text{-}ter}$-mGFP	pHIPN plasmid containing N-terminal region of *PEX23* fused with mGFP under control of *PEX23* promoter; Nat^{R}, Amp^{R}	This study
pHIPH5-*PEX32*-mGFP	pHIPH plasmid containing *PEX32*-mGFP under control of *AMO* promoter; Hph^{R}, Amp^{R}	(Wu et al., 2020)
pHIPN23-*PEX23*$^{N\text{-}ter}$-*PEX32*^{DysF}-mGFP	pHIPN plasmid containing N-terminal region of *PEX23* and DysF region of *PEX32* fused with mGFP under control of *PEX23* promoter; Nat^{R}, Amp^{R}	This study
pARM011	Plasmid containing the *ATG1* deletion cassette; Hph^{R}, Amp^{R}	(Thomas et al., 2018)
pAMK15	pHIPX plasmid containing DsRed-SKL under the control of *TEF* promoter; *LEU2*, Kan^{R}	(Krikken et al., 2009)
pHIPH4 *PEX11*	pHIPH plasmid containing the full length of *PEX11* under the control of *AOX* promoter; Hph^{R}, Amp^{R}	This study

pHIPX4 *PEX11*	pHIPX plasmid containing the full length of *PEX11* under the control of *AOX* promoter; *LEU2*, Kan^R	This study
pHIPX4	pHIPX plasmid with *AOX* promoter; *LEU2*, Kan^R	(Gietl et al., 1994)
pHIPH7 *PEX11*	pHIPH plasmid containing the full length of *PEX11* under the control of *TEF* promoter; Hph^R, Amp^R	This study
pHIPH5-*PEX11*	pHIPH plasmid containing the full length of *PEX11* under the control of *AMO* promoter; Hph^R, Amp^R	This study
pHIPH7-DsRed-SKL	pHIPH plasmid containing DsRed-SKL under the control of *TEF* promoter; Hph^R, Amp^R	(Devarajan et al., 2020)
pSEM04	pHIPH plasmid containing *PEX3* under the control of *AMO* promoter; Hph^R, Amp^R	(Knoops et al., 2014)

Supplementary Table 3: Primers used in this study

Primer	Sequences (5' to 3')
Fw $Pex32_{1-696}$	GCGAAGCTTATGTCTGAGCCCAATGTTCG
Rv $Pex32_{1-696}$	GGAAGATCTGATCTGGAAATCATTGAGCAC
Fw $Pex32_{697-1062}$	GCGAAGCTTATGTCAAATATTGGAACAGG
Rv $Pex32_{697-1062}$	GGAAGATCTGGTGGTTGCGTCGTCCTCG
Rev $Pex32_{1-501}$	GGAAGATCTTCGCGTCATGAGCCAAATG
Rev $Pex32_{1-312}$	GGAAGATCTGTCAATGGTGGTCTTCAAG
Rev $Pex32_{1-177}$	GGAAGATCTAGGATCATCGTTTGTCCAGG
Fw $Pex32_{(169-1062)}$	GCGAAGCTTATGGATGATCCTTATACC
Fw $Pex32_{(94-1062)}$	GCGAAGCTTATGACATCTGCACTGTATGCG
Rv $DysF_{PEX32-mGFP}$	CCGCTCGAGTTACTTGTACAGCTCGTCCATGCC
Fw $Pex32_{o2TM}$	GCGAAGCTTATGGATGATCCTTATACCA
Fw $Pex32_{TM3+4}$	GCGAAGCTTATGGACTTGCGGTCGGAGAC
P_{PEX32} fw	GAATGCGGCCGCCTCGTGGATGTCTTGATAAC
P_{PEX32} rev	CCCAAGCTTAAGAAGAGGTCATAAATGGAG
Fw-Nter-Pex23	CCAGAAGCTTACTTGAAGGACTG
Rv-Nter-Pex23	GGAAGATCTTCCAGCCTTCCTAGTGACC
Fw-P-Pex23	CGCGGATCCACTTGAAGGACTGCTTGGAAAAC
Rv-P-Pex23	CCCAAGCTTGATCGATAGGAAACACAATTTTTC
Fw-Cter-Pex32	GGAAGATCTTCAAATATTGGAACAGGAAAGAC
Rv-GFP-Pex32	CCAATGCATCGATCCTCTAGAGTCGACC
pDEL_ATG1_fwd	ACAGGTCGTTGGTGACTTTAC
pDEL_ATG1_rev	CTTCTCGTTGCCCGTGACC
Pex11-3	CCCAAGCTTATGGTTTGCGACACGATAAC
Pex11-4	AGAGTCGACTCATAGCACAGAAGACTCGG
PEX11-01	TCGAGGATCCATGGTTTGCGACACGATAAC
PEX11-02	CGATCCCGGGTCATAGCACAGAAGACTCGG

REFERENCES

Baerends, R. J., *et al.* (1997). Deviant Pex3p Levels affect Normal Peroxisome Formation in Hansenula polymorpha : high steady-state levels of the protein fully abolish matrix protein import. *Yeast* 13:1437–1448.

Devarajan, S., *et al.* (2020). Proteasome-dependent protein quality control of the peroxisomal membrane protein Pxa1p. *Biochim. Biophys. Acta Biomembr.* 1862(9):183342. doi:

10.1016/j.bbamem.2020.183342

Gietl, C., *et al.* (1994) Mutational analysis of the N-terminal topogenic signal of watermelon glyoxysomal malate dehydrogenase using the heterologous host Hansenula polymorpha. *Proc. Natl. Acad. Sci. U. S. A.* 91(8):3151–5. doi: 10.1073/pnas.91.8.3151

Knoops, K., *et al.* (2014) Preperoxisomal vesicles can form in the absence of Pex3. *J. Cell Biol.* 204(5):659–668. doi: 10.1083/jcb.201310148

Komori, M., *et al.* (1997) The Hansenula polymorpha PEX14 gene encodes a novel peroxisomal membrane protein essential for peroxisome biogenesis. *EMBO J.* 16(1):44–53. doi: 10.1093/emboj/16.1.44

Krikken, A. M., *et al.* (2020) Peroxisome retention involves Inp1-dependent peroxisome-plasma membrane contact sites in yeast. *J. Cell Biol.* 219(10):e201906023. doi: 10.1083/jcb.201906023

Krikken, A. M., *et al.* (2009) Hansenula polymorpha pex11 cells are affected in peroxisome retention. *FEBS J.* 276(5):1429–1439. doi: 10.1111/j.1742-4658.2009.06883.x.

Saraya, R., *et al.* (2012) Novel genetic tools for Hansenula polymorpha. *FEMS Yeast Res.* 12(3):271–8. doi: 10.1111/j.1567-1364.2011.00772.x.

Sudbery, P. E., *et al.* (1988) Hansenula polymorpha as a novel yeast system for the expression of heterologous genes. *Biochem. Soc. Trans.* 16(6):1081-3. doi: 10.1042/bst0161081a.

Thomas, A. S., *et al.* (2018) Hansenula polymorpha Aat2p is targeted to peroxisomes via a novel Pex20p-dependent pathway. *FEBS Lett.* 592(14):2466–2475. doi: 10.1002/1873-3468.13168.

Wu, F., *et al.* (2020) Pex24 and Pex32 are required to tether peroxisomes to the ER for organelle biogenesis, positioning and segregation in yeast. *J. Cell Sci.* 133(16):jcs246983. doi: 10.1242/jcs.246983.
